# Predicting lung function decline in cystic fibrosis: the impact of initiating ivacaftor therapy

**DOI:** 10.1186/s12931-024-02794-2

**Published:** 2024-04-27

**Authors:** Grace C. Zhou, Ziyun Wang, Anushka K. Palipana, Eleni-Rosalina Andrinopoulou, Pedro Miranda Afonso, Gary L. McPhail, Christopher M. Siracusa, Emrah Gecili, Rhonda D. Szczesniak

**Affiliations:** 1 Department of Biostatistics, St. Jude Children’s Research Hospital, Memphis, TN USA; 2 Division of Biostatistics & Epidemiology, Cincinnati Children’s Hospital Medical Center, Cincinnati, OH USA; 3 Biostatistics and Data Management, Medpace, Cincinnati, OH USA; 4 Duke University School of Nursing, Durham, NC USA; 5 Departments of Biostatistics and Epidemiology, Erasmus Medical Center, Rotterdam, The Netherlands; 6 Division of Pulmonary Medicine, Cincinnati Children’s Hospital Medical Center, Cincinnati, OH USA; 7 Department of Pediatrics, University of Cincinnati, Cincinnati, OH USA; 8 Department of Epidemiology, Erasmus Medical Center, Rotterdam, The Netherlands

**Keywords:** CFTR modulators, Cystic fibrosis, Lung function decline, Medical monitoring, Patient registry analysis, Prediction, Predictive probabilities, Rapid decline, Responsiveness, Trajectory

## Abstract

**Background:**

Modulator therapies that seek to correct the underlying defect in cystic fibrosis (CF) have revolutionized the clinical landscape. Given the heterogeneous nature of lung disease progression in the post-modulator era, there is a need to develop prediction models that are robust to modulator uptake.

**Methods:**

We conducted a retrospective longitudinal cohort study of the CF Foundation Patient Registry (*N* = 867 patients carrying the G551D mutation who were treated with ivacaftor from 2003 to 2018). The primary outcome was lung function (percent predicted forced expiratory volume in 1 s or FEV1pp). To characterize the association between ivacaftor initiation and lung function, we developed a dynamic prediction model through covariate selection of demographic and clinical characteristics. The ability of the selected model to predict a decline in lung function, clinically known as an FEV1-indicated exacerbation signal (FIES), was evaluated both at the population level and individual level.

**Results:**

Based on the final model, the estimated improvement in FEV1pp after ivacaftor initiation was 4.89% predicted (95% confidence interval [CI]: 3.90 to 5.89). The rate of decline was reduced with ivacaftor initiation by 0.14% predicted/year (95% CI: 0.01 to 0.27). More frequent outpatient visits prior to study entry and being male corresponded to a higher overall FEV1pp. Pancreatic insufficiency, older age at study entry, a history of more frequent pulmonary exacerbations, lung infections, CF-related diabetes, and use of Medicaid insurance corresponded to lower FEV1pp. The model had excellent predictive accuracy for FIES events with an area under the receiver operating characteristic curve of 0.83 (95% CI: 0.83 to 0.84) for the independent testing cohort and 0.90 (95% CI: 0.89 to 0.90) for 6-month forecasting with the masked cohort. The root-mean-square errors of the FEV1pp predictions for these cohorts were 7.31% and 6.78% predicted, respectively, with standard deviations of 0.29 and 0.20. The predictive accuracy was robust across different covariate specifications.

**Conclusions:**

The methods and applications of dynamic prediction models developed using data prior to modulator uptake have the potential to inform post-modulator projections of lung function and enhance clinical surveillance in the new era of CF care.

**Supplementary Information:**

The online version contains supplementary material available at 10.1186/s12931-024-02794-2.

## Background

Cystic fibrosis (CF) is a progressive, genetic disease caused by CF transmembrane conductance regulator (CFTR) protein dysfunction, leading to cyclical lung infection and inflammation. As a result, lung function monitoring through longitudinal measurement of forced expiratory volume in 1 s of % predicted (FEV1pp) has been a key element of CF care. Highly effective CFTR modulator therapies, which are designed to correct malfunctioning protein made by the CFTR gene, have revolutionized the clinical landscape. The first of these therapies, ivacaftor, which has been in widespread use in the U.S. since January 2012 for select mutations (e.g., G551D), has been shown to improve FEV1pp markedly [1]. Analysis of trial participants with a G551D mutation on ivacaftor, matched with historical F508del homozygotes from the U.S. Cystic Fibrosis Foundation Patient Registry (CFFPR) as controls, found that ivacaftor treatment associated with 50% slower decline in FEV1pp over a 3-year period [2]. A more recent pre-post study of the Canadian CF Registry considered follow-up as long as 8 years before and 8 years after ivacaftor initiation and reported more variable benefits, demonstrating age-related reductions in FEV1pp slope of 60% and 71% for pediatric and adult ivacaftor patients, respectively (difference in the FEV1pp slope between the pre and post ivacaftor: 0.58 predicted/year and 0.72 predicted/year, respectively) [3]. Another study of CF patients who carry the G551D mutation included data from a two-year clinical trial and a 5-year observational cohort from West of Scotland suggested sustained lung function benefit from ivacaftor use among adults, but long-term improvement seemingly plateaued year over year in the pediatric population [4]. These findings corroborate a prior U.S. multi-center study, which showed that the rate of decline in FEV1pp over a 5.5-year period after ivacaftor initiation was worse for pediatric subjects than adults (1.68% predicted/year versus 0.63% predicted/year) [5].

While treatment heterogeneity is expected, the reported variability, both between patients and within an individual patient over time, demonstrates the need to reevaluate the suitability of both FEV1pp prediction models and the thresholds used to identify meaningful lung function decline after ivacaftor initiation. Prediction models of CF FEV1pp decline have historically been useful for early identification of pulmonary exacerbation events and other phenomena clinically known as “rapid decline,” which have been defined as a meaningful drop in FEV1pp relative to center- and/or patient-level norms [6, 7]. Early detection and timely treatment of rapid decline improve lung function but can be difficult to achieve without prediction models [8–10]. One such model was developed specifically for real-time prediction of rapid decline in CF using target functions [10]. These functions were derived mathematically based on clinically relevant definitions of rapid decline. However, these definitions reflect pre-modulator thresholds (e.g., drops more than 1.5% predicted/year) [11]. More recent approaches have included the use of an alternative method to identify pulmonary exacerbations, known as the FEV1-indicated exacerbation signal (FIES) [12]. While pulmonary exacerbation has historically been defined heterogeneously through assessment primarily of lung function drops, there has been limited consensus on how to define such drops [13].

For these reasons, the aims of our study were to (i) evaluate the robustness of an existing prediction model framework that accurately detected rapid decline in the pre-modulator era and (ii) adapt this framework to examine a novel target function specific to FIES events.

## Methods

### Study design and cohort

We conducted a retrospective longitudinal cohort study using the CFFPR, which is a national patient registry that collects demographic and clinical data on individuals with CF who are patients at care centers across the U.S [14]. The study included data from 2003 to 2018. To construct the analysis cohort, we considered patients with a valid CF diagnosis (e.g., blood test, sweat test, genetic test) who carry the G551D mutation and received ivacaftor any time after January 1, 2012, which represents the era of widespread U.S. Food and Drug Administration (FDA) approval for this therapy. Data observed when patients were younger than 6 years old was excluded, given the potential for unreliable pulmonary function test (PFT) results in very young patients with CF. Data observed after lung transplantation were censored.

**Fig. 1:**
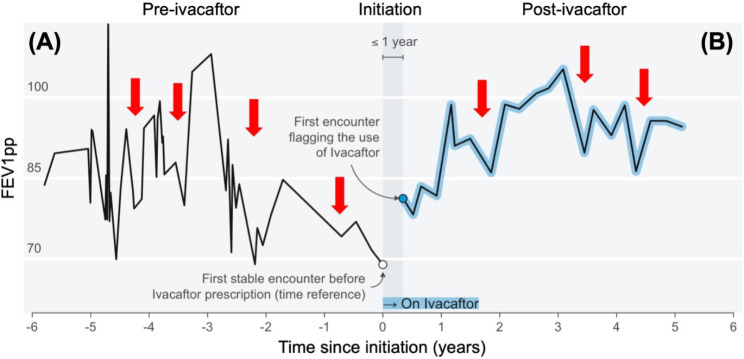
Lung function trajectory and ivacaftor initiation shown for a male with cystic fibrosis in the analysis cohort. The outcome was measured as the percent predicted forced expiratory volume in 1 s (FEV1pp, y-axis) observed over time (x-axis). (A) before and (B) after ivacaftor initiation. His pre-ivacaftor FEV1 ranged from 68 to 118% predicted, while post-ivacaftor was 82 to 102% predicted. His overall FEV1pp increased, but he experienced FIES events (examples of events shown using red arrows in A and B) before and after ivacaftor initiation

*Determining ivacaftor initiation.* We omitted PFTs observed during the first 30 days after the CFFPR-recorded ivacaftor start date (Fig. 1). Our rationale was (i) we wanted to avoid estimating the initial increase in FEV1pp related to ivacaftor that has been previously reported in other analyses [2], and (ii) we sought to reduce the potential bias between the ivacaftor prescription date and the actual start date recorded in the CFFPR. To ensure valid estimation before and after ivacaftor initiation, we restricted the cohort to patients who had at least one PFT before initiation and at least two PFTs after, and for whom the earliest and latest post-initiation PFTs were separated by at least 6 months in time.

*FIES definition*. We derived our criteria at each clinical encounter from the definition provided by the CF Learning Network [12], which was applied as follows:

1. For baseline FEV1pp ≥ 50, if the current FEV1pp represents a 10% or more relative decline in lung function, compared to the baseline.
2. For baseline FEV1pp < 50, if the current FEV1pp represents a 5% or more relative decline in lung function, compared to the baseline.

In this definition, baseline is the average of the two highest FEV1pp values in the past 12 months that were not recorded during intravenous antibiotic treatment. Expanded details on the FIES definition are provided as supplemental material (Part 1, Section I).

### Statistical analysis

*Outcome, covariates, and missing data.* The study outcome was the FEV1pp value observed at each clinical encounter. Covariates included observed demographic and clinical characteristics that have been previously associated with accelerated FEV1pp decline: the time-varying variables were Medicaid insurance use, infection with methicillin-resistant *Staphylococcus aureus* (MRSA), infection with *Pseudomonas aeruginosa* (Pa), diagnosis of CF-related diabetes (CFRD), and numbers of acute pulmonary exacerbations and outpatient visits within the previous year; the non-time-varying variables were age and FEV1pp at study entry, birth cohort (defined based on year of birth), sex, and pancreatic insufficiency (defined as any reported use of pancreatic enzymes). All subsequently described prediction modeling assumed that outcome data were missing at random [15]. 

*Prediction model setup.* A previously described longitudinal model framework with nonstationary stochastic process to the prediction of FEV1pp and the use of clinically relevant target functions in CF was adapted for this study [10]. Particularly, the variance terms in the linear mixed effects model included a random intercept to account for between-patient variation, an integrated Brownian motion covariance function to account for within-patient variation over time and to allow us to create predictive probability distributions using a prior approach, and a residual measurement error [16]. We first set up a saturated model within this framework to examine various covariate effects. The time since study entry (in years) was used as the time variable. A change point term, which represented pre- and post-ivacaftor initiation periods, was included as a main effect, and its interaction with time was used to examine associations between ivacaftor response and absolute FEV1pp, as well as the difference in slopes between pre- and post-ivacaftor initiation periods. Covariates were considered as both main effects and interactions with the time variable in the saturated model. Results were scaled to the time since ivacaftor initiation (in years) for presentation purposes. Details of the model setup are presented as supplemental material (Part 2, Section I) and the residual diagnostics of the selected model are shown in the supplemental material (Part 1, Section II).

*Covariate selection.* Reduced forms of the previously described terms in the saturated model were examined with the Akaike and Bayesian information criteria (AIC and BIC, respectively) and the likelihood ratio test (LRT).

*Predictive probabilities for FIES events.* The target function derived from the above FIES definition was implemented as part of the model fitting in the R package “lmenssp” version 1.2 [17]. The formulas and code can be found from supplementary material Part 2, Sections II-III and Part I, Section V, respectively.

**Fig. 2:**
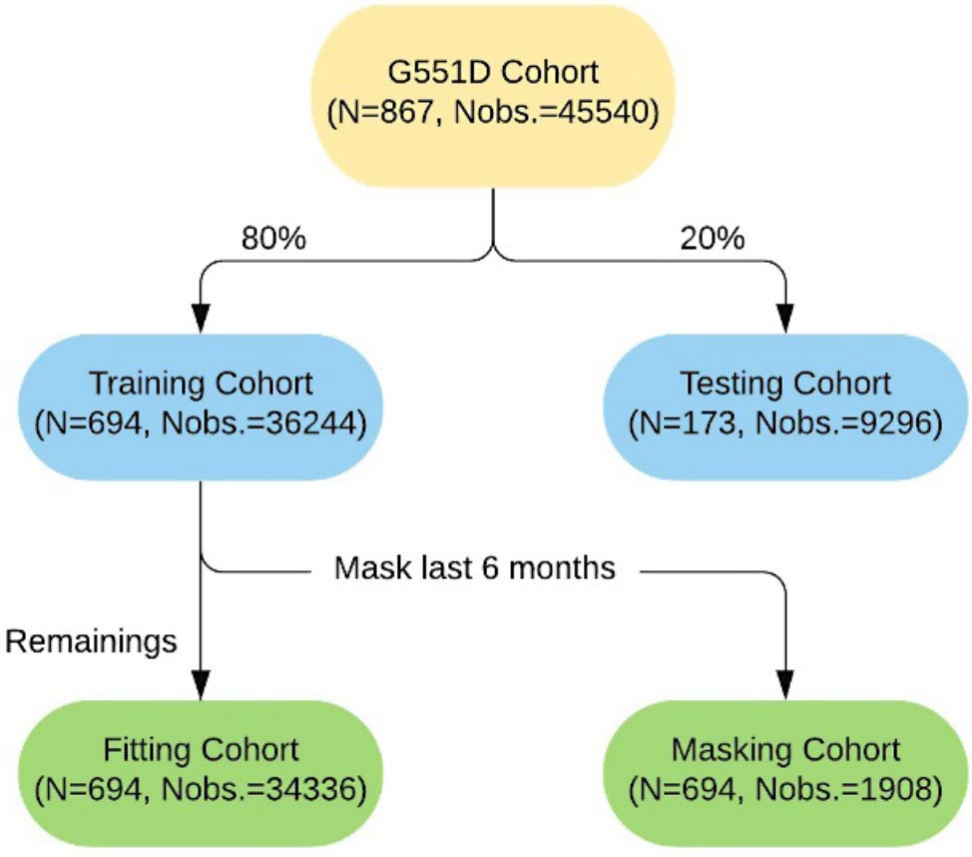
Dataflow for model fitting and validation. The overarching analysis cohort (the first level) was segmented into training and testing cohorts (the second level). The training cohort was further split into a cohort for model fitting and a masked cohort for forecast validation (the third level)

*Validation.* Two types of validation were performed that are relevant to clinical scenarios: (i) predictions for “new patients,” and (ii) forecasting for patients who were part of the model building but return for follow-up visits (i.e., updated predictions). To accomplish both types of predictions, the analysis cohort was randomly split into 80% for training and 20% for independent testing (Fig. 2). For type (i), we examined predictions within the testing cohort. For type (ii), we examined data that were held out for the last 6 months of follow-up in the training cohort. Both types of predictions were evaluated using 5-fold cross-validation.

*Evaluation of predictive performance.* Metrics to evaluate predictive accuracy included the root-mean-square error (RMSE), the mean absolute error (MAE), the Brier score, and the area under the receiver operating characteristic curve (AUC). Formulas for the metrics are provided as supplemental material (Part 1, Section III). Lower values of the RMSE, MAE, and Brier score imply higher predictive accuracy, while lower AUC values indicate lower predictive accuracy. The 95% confidence interval (CI) for each AUC estimate was obtained through nonparametric bootstrapping with 1,000 replicates via the R package “boot” version 1.3–28.1 [18]. 

## Results

### Patient characteristics

The analysis cohort consisted of 867 ivacaftor-treated patients with CF who had a total of 45,540 PFTs over the study timeframe. Patients typically entered the study as adolescents with ivacaftor initiation in early adulthood (Table 1). There were slightly more males than females (53.1% versus 46.9%). Most patients had pancreatic insufficiency, reported Medicaid insurance use, and had lung infections with MRSA or Pa during the follow-up period. Slightly more than half of all patients developed CFRD. There was a total of 11,328 FIES events, which occurred in 27% and 25% of observations before and after ivacaftor initiation, respectively.

**Table 1:** Characteristics of Ivacaftor-Treated Cohort*

Characteristics	Total Number of Patients (*N* = 867)
Age at entry	14.7 (6.00–68.5)
Age at ivacaftor initiation	21.6 (6.19–68.7)
FEV1pp at entry	83.7 (18.0–140)
**Sex**	
Female	407 (46.9%)
Male	460 (53.1%)
Birth Cohort	
<1968	37 (4.3%)
[1968,1982)	131 (15.1%)
[1982,1996)	377 (43.5%)
>1996	322 (37.1%)
Pancreatic insufficiency	828 (95.5%)
**Medicaid insurance use**	
At entry	371 (42.8%)
Ever during follow-up	763 (88.0%)
MRSA infection	
At entry	65 (7.5%)
Ever during follow-up	490 (56.5%)
Pa infection	
At entry	205 (23.6%)
Ever during follow-up	699 (80.6%)
**CFRD**	
At entry	38 (4.4%)
Ever during follow-up	449 (51.8%)
Duration of follow-up per patient	12.36 (1.04–15.96)
Number of observations over follow-up per patient	48 (5–204)

*Entry refers to the first available clinical encounter for each patient in the analysis cohort. Continuous and categorical variables are summarized as mean (minimum – maximum) and frequency (%), respectively). Abbreviations: CFRD = cystic fibrosis-related diabetes; FEV1pp = percent predicted forced expiratory volume in 1 s; MRSA = methicillin-resistant *Staphylococcus aureus*; Pa = *Pseudomonas aeruginosa*

### Prediction model

**Fig. 3:**
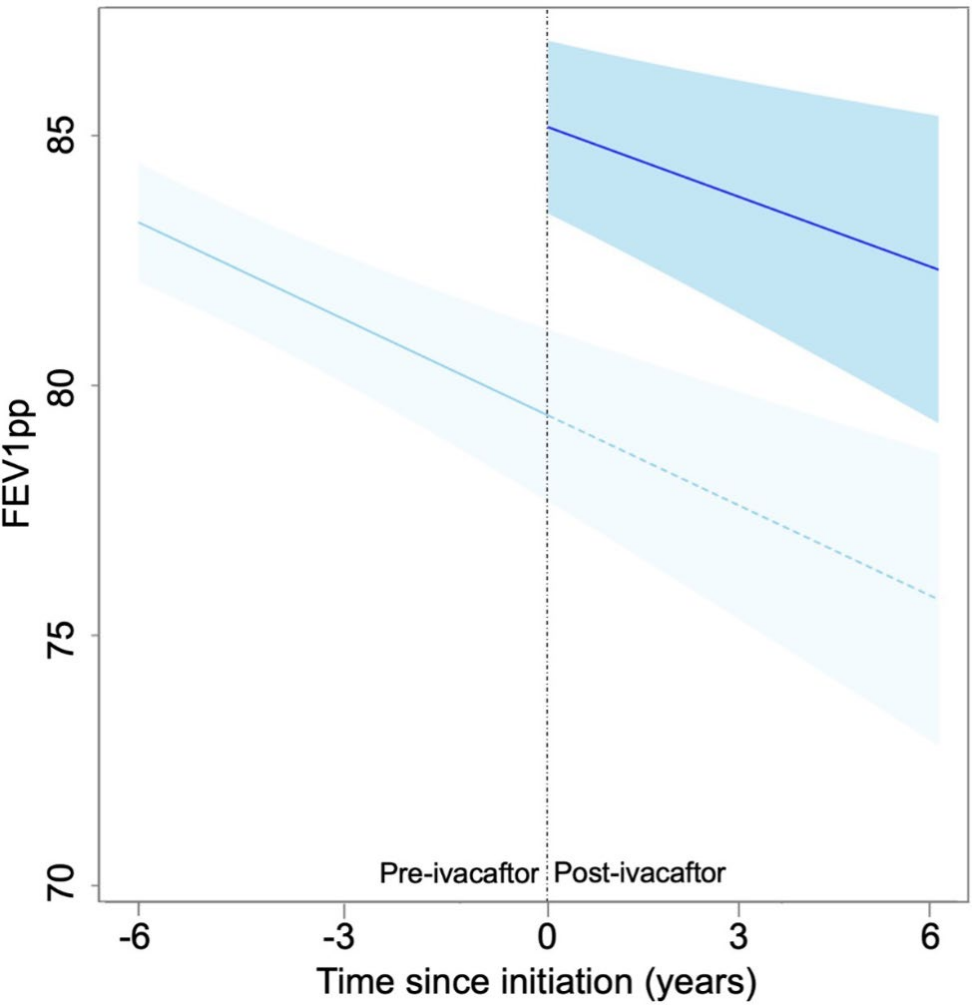
Population-level estimate of lung function trajectories and 95% confidence bands for the pre-ivacaftor trend (lightly shaded curve, extended to show the projected rate of decline without ivacaftor initiation) versus the post-ivacaftor trend (darker shaded curve). The outcome was measured as the percent predicted forced expiratory volume in 1 s (FEV1pp, y-axis) observed over the follow-up time (in years, x-axis). The start of the darker blue curve (vertical dashed line) corresponds to ivacaftor initiation

In the final model, ivacaftor initiation was associated with a change in absolute FEV1pp and a slower rate of decline in FEV1pp (Table 2). The estimated improvement in FEV1pp was 4.89% predicted (95% CI: 3.90 to 5.89). The rate of decline was reduced by 0.14% predicted/year (95% CI: 0.01 to 0.27). The overall rate of FEV1pp decline throughout the follow-up period was 0.61% predicted/year (95% CI: 0.84 to 0.37). Those who had more outpatient visits before entering the study and males tended to have higher absolute FEV1pp values. Pancreatic insufficiency, older age at study entry, a history of more frequent pulmonary exacerbations prior to the study, infection with MRSA or Pa, CFRD, and use of Medicaid insurance corresponded to lower absolute FEV1pp values. In model selection, these covariates were not associated with an accelerated rate of decline in FEV1pp. The final model also indicated high between-patient variability (quantified as the estimated standard deviation [SD] and 95% CI, which were 8.10 and 7.59 to 8.57, respectively). The changes in absolute FEV1pp corresponded to a meaningful increase and slower decline after ivacaftor initiation (Fig. 3). Carrying forward the projected rate of decline from the pre-ivacaftor initiation period (the light blue dashed line), there were differences between the pre- and post-ivacaftor initiation trajectories with non-overlapping 95% CIs (comparing the light blue dashed line with the darker blue solid line during the post-ivacaftor period).

**Table 2:** Parameter Estimates from Ivacaftor-Informed Prediction Model

Parameters*	Estimate	95% CI	SE	P
Fixed effect terms				
Intercept	82.58	(76.81, 88.35)	2.94	0.00
Time since study entry	-0.61	(-0.84, -0.37)	0.12	0.00
Change point, pre- to post-ivacaftor initiation	4.89	(3.90, 5.89)	0.51	0.00
Time × change point**	0.14	(0.01, 0.27)	0.07	0.04
FEV1pp at study entry	0.74	(0.71, 0.78)	0.02	0.00
Age at study entry	-0.16	(-0.32, 0.00)	0.08	0.05
Frequency of acute pulmonary exacerbations within prior year	-0.23	(-0.28, -0.18)	0.03	0.00
Number of outpatient visits within prior year	0.23	(0.17, 0.29)	0.03	0.00
Pa infection	-1.26	(-1.53, -0.99)	0.14	0.00
MRSA infection	-1.54	(-1.89, -1.18)	0.18	0.00
CFRD	-0.95	(-1.34, -0.56)	0.20	0.00
Male (Ref: female)	0.16	(-1.14, 1.46)	0.66	0.81
Pancreatic insufficiency	-0.18	(-0.44, 0.09)	0.14	0.19
Medicaid insurance usage	-0.26	(-0.6, 0.07)	0.17	0.12
Born 1982–1996 (Ref: born 1968–1982)	1.15	(-1.93, 4.23)	1.57	0.47
Born < 1968	0.85	(-3.47, 5.16)	2.20	0.70
Born > 1996	2.45	(-1.57, 6.48)	2.05	0.23
Variance terms				
Between patient variability	65.58	(57.67, 73.48)	4.03	
Within patient variability	5.16	(4.78, 5.54)	0.19	
Residual variability	67.65	(66.58, 68.71)	0.54	

*Coefficient estimates of fixed effects should be combined across other covariates to find average estimate of absolute FEV1pp. Positive estimates for main effects terms correspond to increase in FEV1pp. Positive estimates for interaction terms imply increase in slope of FEV1pp (i.e., less rapid decline). Abbreviations: CI=confidence interval; CFRD = cystic fibrosis related diabetes; FEV1pp = forced expiratory volume in 1 s of % predicted; MRSA = Methicillin resistant Staphylococcus aureus; Pa = Pseudomonas aeruginosa

### Predictive accuracy

The selected model presented a reasonable predictive performance for both the FEV1pp outcome and FIES events (Table 3). It achieved similar predictive accuracy for FEV1pp across the different segments of the analysis cohort, which were formed to carry out the different types of validation, indicating the ability of the model to predict data in real time and to forecast data accurately. In the case of FIES events, the Brier Score consistently remained low across all cohorts, which are indicative of better-calibrated predictions. The AUC (over 80%) indicated high levels of predictive accuracy, with the highest accuracy in the masked cohort and similar levels of accuracy in the fitting and independent testing cohorts.

**Table 3:** Predictive performance of the model*

	FEV1pp		FIES	
Cohort (N)	RMSE (SD)	MAE (SD)	Brier Score (SD)	AUC (95% CI)
Fitting cohort(*N* = 694)	7.34 (0.08)	5.19 (0.06)	0.14 (0)	0.83 (0.83,0.83)
Masking cohort (*N* = 694)	6.78 (0.20)	4.48 (0.1)	0.12 (0)	0.90 (0.89,0.9)
Testing cohort (*N* = 173)	7.31 (0.29)	5.16 (0.21)	0.14 (0.01)	0.83 (0.83,0.84)

*Average values obtained from 5-fold cross-validation, including standard deviation shown for RMSE, MAE, and Brier scores. Abbreviations: CI=confidence interval; FEV1pp = forced expiratory volume in 1 s of % predicted; FIES = FEV1-Indicated Exacerbation Signal

### Dynamic predictions

**Fig. 4:**
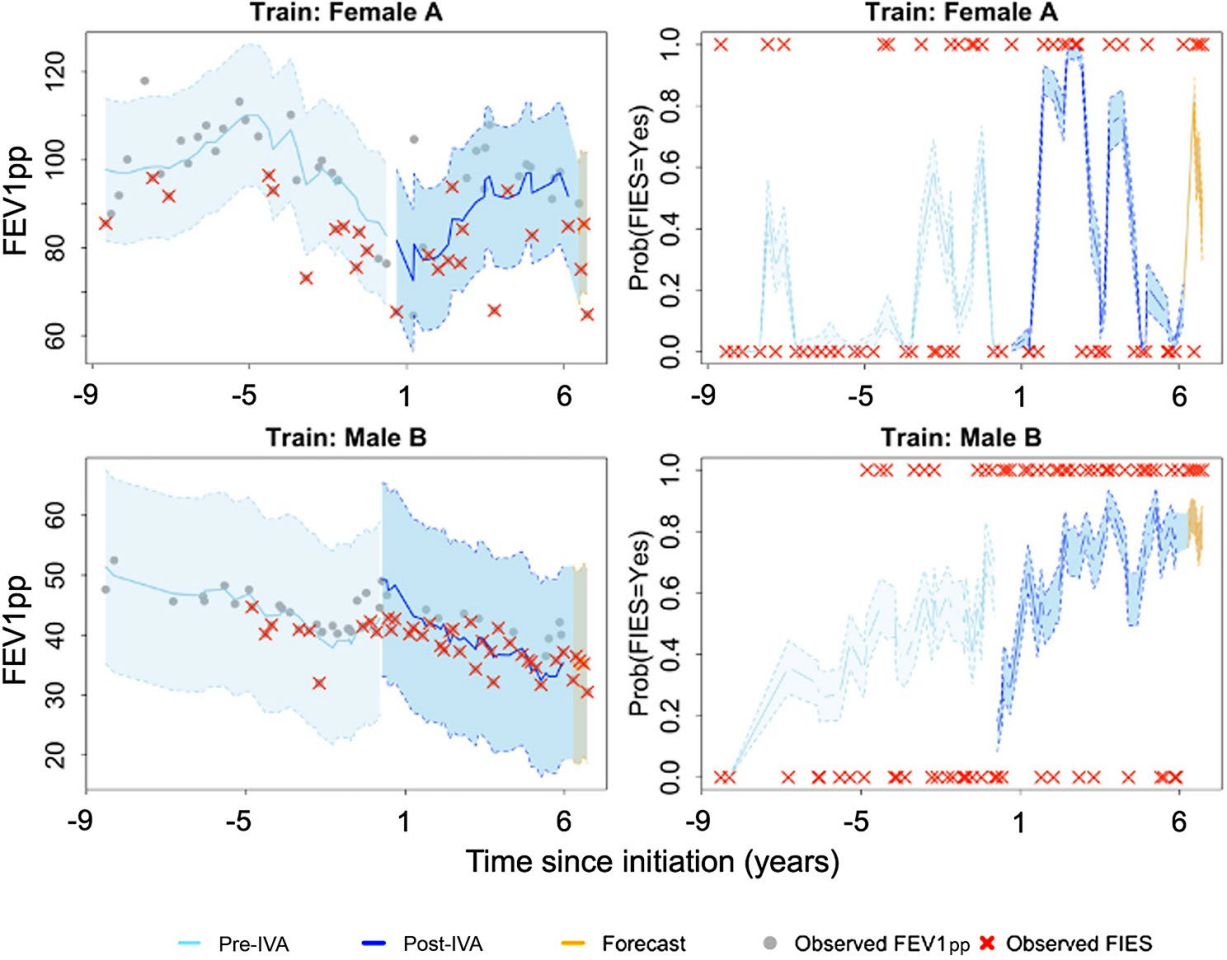
Dynamic predictions from the training cohort shown for a female patient with baseline age 9.36 years (first row) and a male patient with baseline age 31.2 years (second row). Left panel: observed FEV1pp against time with estimated values, 95% CI, and six-month forecasted lung function; gray shaded area shows estimation for 6-month held-out data. Right panel: predictive probabilities of FIES, including observed FIES events, predicted probability, and bootstrapped 95% CI. Abbreviations: CI = confidence interval; IVA = ivacaftor

To further illustrate the model’s predictive performance at an individual patient level, we evaluated the predictive probability of FIES events for two randomly selected patients from the training set (Fig. 4). The top row shows a female CF patient. Her trajectory was variable throughout both the pre- and post-ivacaftor initiation periods (represented by light blue and dark blue shaded trajectories, respectively). She experienced a series of FIES events over both periods. The gray area shows the 95% confidence band for projected estimates of FEV1pp and the trend during the 6-month held-out timeframe. Her predictive probability of FIES events also varied significantly during both the pre- and post-ivacaftor initiation periods. The model accurately projected an elevated risk of FIES events during periods in which they occurred, including in the yellow-shaded prediction intervals for the 6-month held-out timeframe. The male CF patient in the bottom row had a more stable FEV1pp trajectory that exhibited more gradual increases, with a FIES risk that increased over time.

**Fig. 5:**
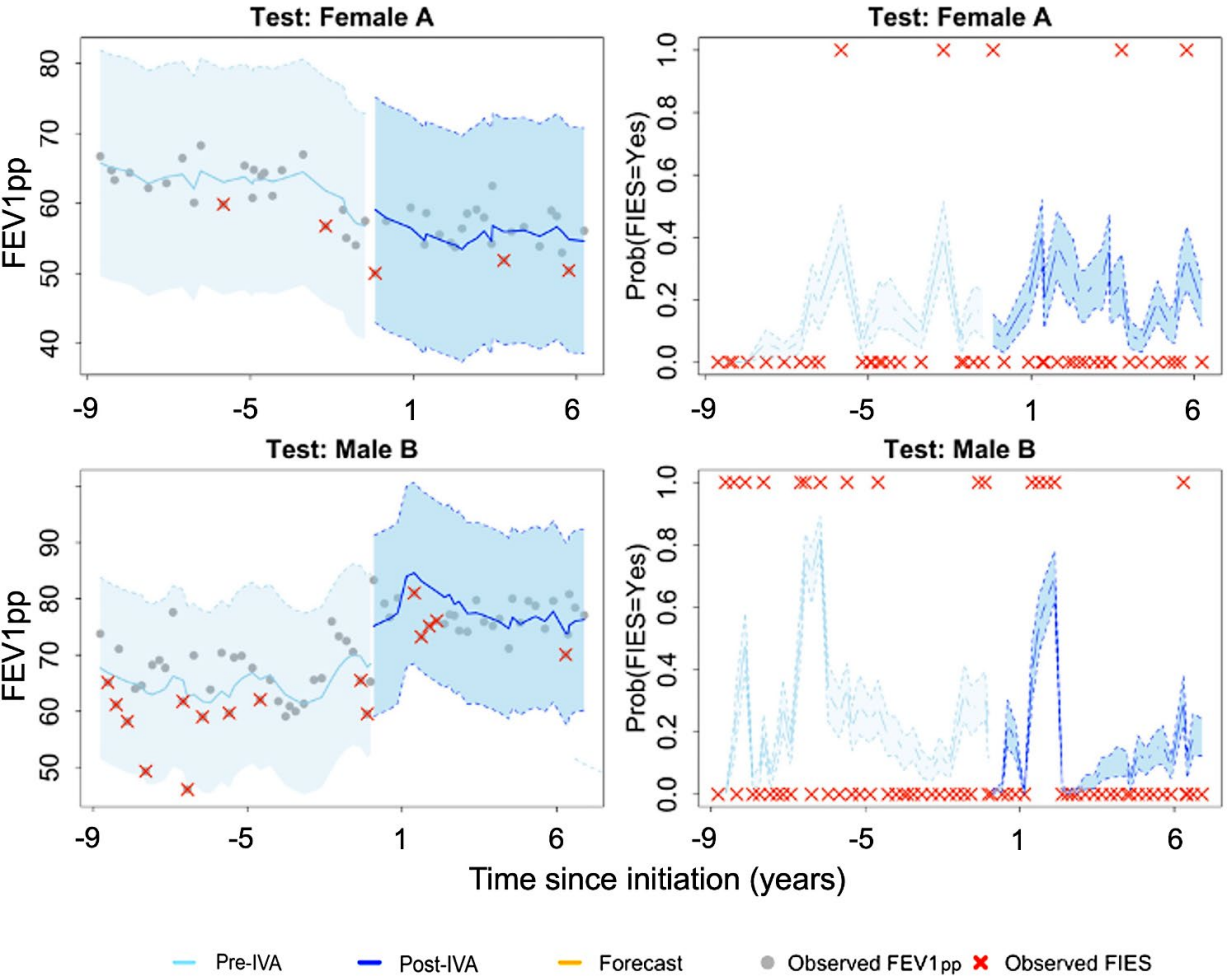
Dynamic predictions from the independent test validation cohort for a female patient with baseline age 48.8 years (first row) and a male patient with baseline age 15.4 years (second row). Left panel: observed FEV1pp against time with estimated values, 95% CI, and six-month forecasted lung function. Right panel: predictive probabilities of FIES, including observed FIES events, predicted probability, and bootstrapped 95% CI. Abbreviations: CI = confidence interval; IVA = ivacaftor

We similarly showed individual trajectories for patients who were in the independent testing cohort (Fig. 5). The female CF patient with data and predictions displayed in the top row experienced a more gradual FEV1pp decline over the follow-up period with relatively few FIES events. Although this patient did not contribute data to the model development, since she was in the testing cohort, her projected low risk of FIES was accurate (based on the cut point for predictive probabilities from the receiver operating characteristic analysis). The male CF patient in the bottom row appears to have benefited from ivacaftor initiation, as evidenced by the overall increase in FEV1pp and slower rate of FEV1pp decline.

### Robustness of predictive performance across models

The individual predictive performance of the models tended to be invariant under different combinations of covariates. To evaluate the robustness of predictive performance across models, we performed simulation studies that support this conclusion (see supplemental Part 1, Section IV a, for further details). From the results, we found that to choose the “best” model, we should rely on model information criteria (AIC and BIC) rather than accuracy metrics because in some cases, both correctly and incorrectly specified models presented similar, small predictive errors, but their AIC and BIC values were distinct. In addition, in cases with extremely poor model performance (i.e., extremely large RMSE or AIC/BIC), we found that examining different variance-covariance structures improved performance. Across these alternative models, all those with the ivacaftor initiation period as a covariate had both higher AIC and BIC values (see Supplement Part 1, Section IV b).

## Discussion

This study found that dynamic prediction models from the pre-CFTR modulator era can be effectively adapted to characterize ivacaftor responsiveness in people with CF while providing accurate, individualized predictions of precipitous drops in lung function that may provide an early signal for the onset of a pulmonary exacerbation. We developed a novel target function to predict FIES events, which serve as data-driven surrogates intended to standardize pulmonary exacerbation definitions and enhance early detection. Moreover, the findings demonstrate that FIES events were equally prevalent before and after ivacaftor initiation, highlighting the need for lung function monitoring even after a modulator is initiated.

Parameter estimates from this model echoed the short-term ivacaftor benefits on FEV1pp that were observed in prior studies, along with the potential for dwindling effects. While we observed a 23% reduction in the rate of FEV1pp decline with ivacaftor initiation, this reduction was less substantial than in the Canadian CF registry study, which included a wider variety of mutation types [3]. However, the lack of consensus on a minimal clinically important difference for FEV1pp as a CF clinical trial endpoint makes it challenging to determine the clinical relevance of observed differences [19]. Another prospective multi-center study that included only G551D patients treated with ivacaftor found an average post-ivacaftor initiation decline of 1.22% predicted/year over the 5.5-year study period, which is steeper than our current study estimate. Despite these differences, which may be attributable to differences in cohort definitions, covariate information, or model structure, the dynamic prediction model developed in the current study was able to estimate lung function trajectory and FIES-defined drops accurately at the individual patient level. Perhaps more importantly, the current study sought to develop an accurate prediction model that allows projections after initiation rather than estimating ivacaftor effects; explaining and predicting are viewed in statistical modeling as requiring two different approaches [20]. For the purposes of real-time lung function monitoring and clinical surveillance, the dynamic prediction model and predictive probabilities of FIES can be embedded into existing prediction tools that have relied on frameworks established prior to the availability of CFTR modulators [21]. 

Although the current study focused only on ivacaftor use among individuals with a G551D mutation, highly effective modulator therapy is now available for many CF patients with other ivacaftor-responsive variants (10–15% of patients) and/or a copy of the most common CFTR variant, F508del corresponding to treatment with elexacaftor/tezacaftor/ivacaftor therapy (currently totaling 94% of the U.S. population, given latest FDA approvals) [22–24]. Available short-term clinical effectiveness studies of elexacaftor/tezacaftor/ivacaftor suggest substantial improvements over a 6-month period (increase in FEV_1_: 9.76 [8.76, 10.76]% predicted) [25–27]. These studies also indicated response heterogeneity within subgroups (the improvement ranged from 6.14 to 10.84% predicted depending on prior use of another modulator). Challenges to evaluating the robustness of prediction models for patients on elexacaftor/tezacaftor/ivacaftor include limited post-approval follow-up and less frequent PFTs during 2020 and part of 2021, which could be attributed to COVID-19 pandemic restrictions or the positive pulmonary impacts of the therapy itself [28]. 

While our retrospective analysis provides valuable insights, it is essential to acknowledge the absence of prospective validation. The broad uptake of the newest modulator, elexacaftor/tezacaftor/ivacaftor, which coincided with the COVID-19 pandemic era, brings about the need for assessing its effectiveness in new data and mitigating the risk of overfitting to historical data. Future work should prioritize prospective validation and adjust our approach based on its performance in practical settings to account for other modulator use and the pandemic. Our study uses an accurate prediction model to identify FIES events, which correspond to a universal definition of what constitutes a decline in FEV1pp. However, real-world diagnoses of pulmonary exacerbations may consider changes in symptoms or other clinical factors, such as weight. A symptom-indicated exacerbation score has also been considered, as well as standardizing home exacerbation detection [12]. The prediction model framework presented here could be extended to incorporate weight thresholds, e.g., changes in body mass index over time, as another element of the target function. More novel statistical methodology is required to implement bivariate target functions, but this represents an important research direction for clinical monitoring in the post-modulator era.

## Conclusions

The methods and applications of dynamic prediction models developed using pre-CFTR modulator data have the potential to inform post-CFTR modulator projections of lung function and enhance clinical surveillance in the new era of CF.

### Electronic supplementary material

Below is the link to the electronic supplementary material.

Supplementary Material 1

## Data Availability

The data that support the findings of this study are available from the Cystic Fibrosis Foundation, but restrictions apply to the availability of these data, which were used under an information use agreement for the current study, and so are not publicly available. Requests for these data can be directed to the Cystic Fibrosis Foundation Patient Registry Team via email: datarequests@cff.org.

## Abbreviations

AUC: Area under the receiver operating characteristic curve
CF: Cystic fibrosis
CFFPR: Cystic Fibrosis Foundation Patient Registry
CFRD: Cystic fibrosis-related diabetes
FEV1pp: Percent predicted forced expiratory volume in 1 s
FIES: FEV1-indicated exacerbation signal
MAE: Mean absolute error
MRSA: Methicillin-resistant *Staphylococcus aureus*
Pa: *Pseudomonas aeruginosa*
RMSE: Root-mean-square error
SD: Standard deviation
