# Supplementary material for: Predicting lung function decline in cystic fibrosis: the impact of initiating ivacaftor therapy
Source: Respir Res. 2024 Apr 27;25:187. doi: 10.1186/s12931-024-02794-2 (PMC11056050; doi:10.1186/s12931-024-02794-2)
Supplement: Supplementary file 1 — Supplementary Material 1 [file 12931_2024_2794_MOESM1_ESM.pdf]

Online Supplement (Parts 1-2):

Predicting Lung Function Decline in Cystic Fibrosis: Impact of Initiating Ivacaftor Therapy

Grace C. Zhou, Ziyun Wang, Anushka Palipana, Eleni-Rosalina Andrinopoulou, Pedro M. Afonso[5], Gary L. McPhail, Christopher Siracusa, Emrah Gecili, and Rhonda D. Szczesniak

Contents of Part 1

I. Definition of FIES 3

A. Rolling baseline 3

B. FIES 3

II. Residual plots 4

III. Predictive metrics 5

IV. Sensitivity analyses 6

A. Simulation 6

B. Alternative models 7

1. Model information criteria 7

2. Likelihood ratio test 7

V. Example Code 8

I. Definition of FEV1-indicated exacerbation score (FIES)

A. Rolling baseline

Average of highest two FEV1 values in past 12 months that were not recorded during IV antibiotic treatment:

- Two highest FEV1 values could be ties
- If only one valid FEV1 value, it is used as rolling baseline value
- If no value in past 12 months, there is no rolling baseline value

B. FIES

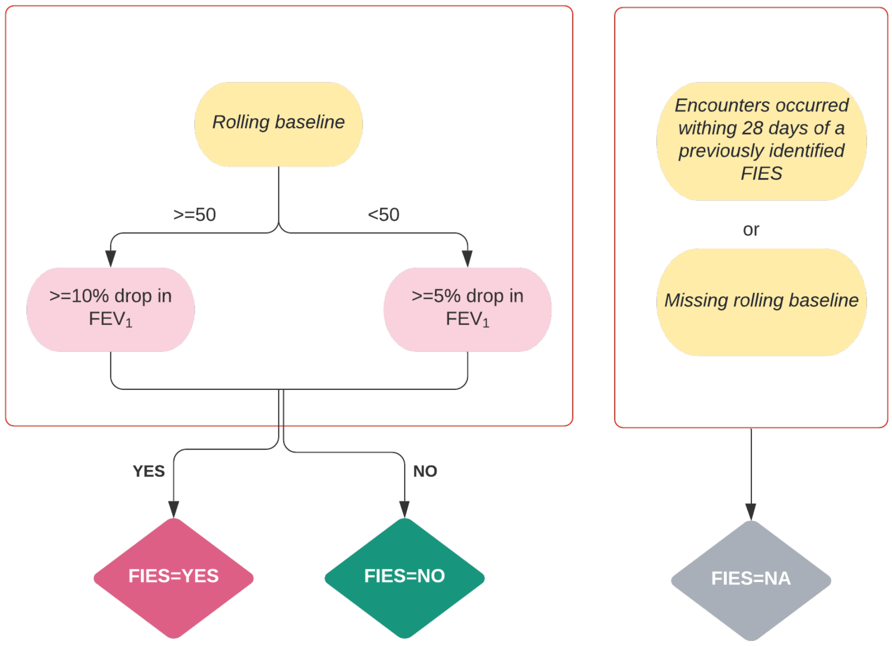

Figure 1.Definition of FIES

II. Residual plots

Conditional standardized residuals are adapted to the rationale from (Szczesniak et al., 2020). Figure 2 illustrates no striking violations of model assumptions, despite quantile-quantile plot implies heavier tails than the standard normal distribution.

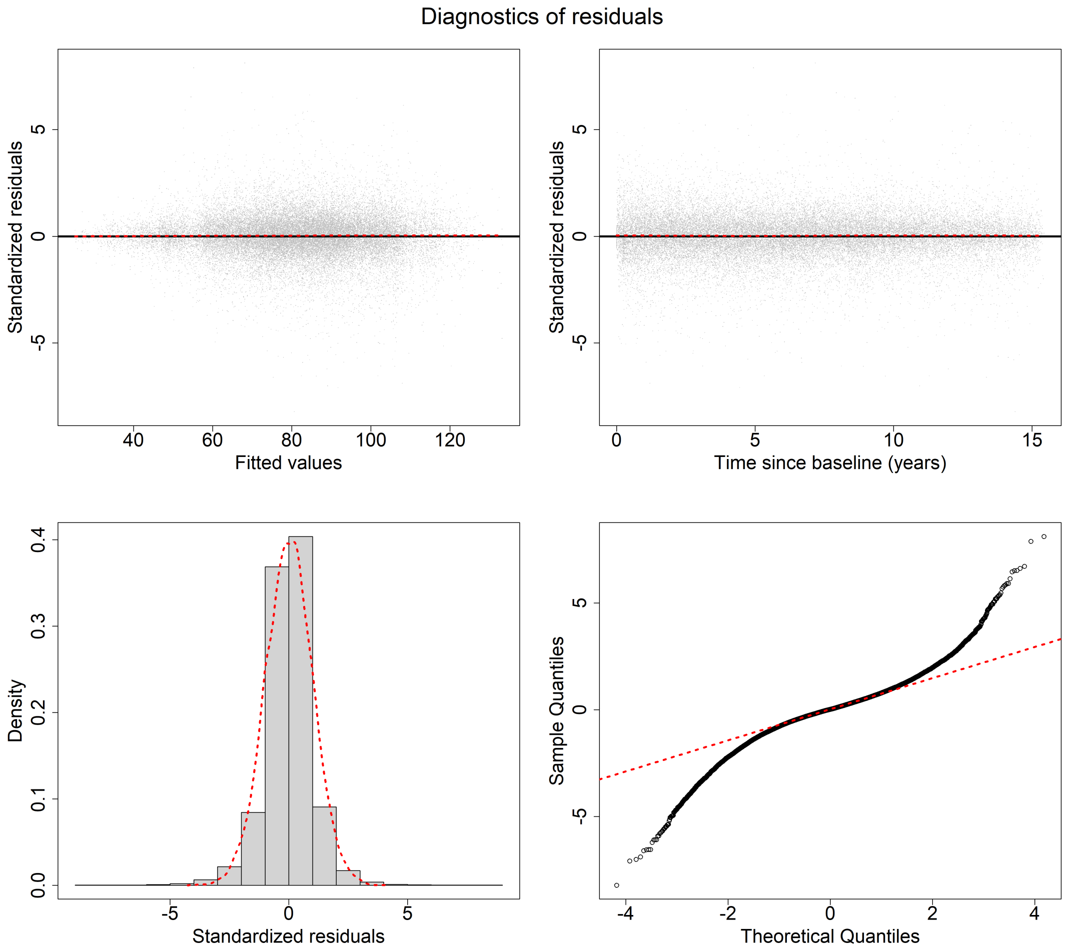

Figure 2.Residuals plot. Upper panel: subject-specific standardized residuals versus fitted values and time; Lower panel: Density and normal Q-Q plot of standardized residuals

III. Predictive metrics

Metrics	Formula	Note
**Root Mean Squared Error (RMSE)**	$\sqrt{\frac{\sum_{i=1}^{n}\sum_{j=1}^{n_i}\left(\hat{y}_{ij}-y_{ij}\right)^2}{\sum_{i=1}^{n} n_i}}$	A small RMSE indicates high prediction accuracy
**Mean absolute Error (MAE)**	$\frac{\sum_{i=1}^{n}\sum_{j=1}^{n_i}\lvert\hat{y}_{ij}-y_{ij}\rvert}{\sum_{i=1}^{n} n_i}$	A small MAE indicates high prediction accuracy
**Brier Score (Brier)**	$\frac{\sum_{i=1}^{n}\sum_{j=1}^{n_i}\left(p_{ij}-o_{ij}\right)^2}{\sum_{i=1}^{n} n_i}$ where p_{ij} denotes predicted probability, o_{ij} denotes observed value out of (0,1)	A small Brier Score indicates high prediction accuracy. Refer to (Brier, 1950)
**Receiver operating characteristic-Area Under Curve (ROC-AUC)**	**X-axis**: 1-specificity=false positive fraction=FP/(FP+TN); **Y-axis**: sensitivity=true positive fraction=TP/(TP+FN); **AUC**: $(\mathrm{X_k}-\mathrm{X_{k-1}})$ * $(\mathrm{Y_k}-\mathrm{Y_{k-1}})/2$	The closer an ROC curve is to the upper left corner (or larger AUC), the more efficient is the test.(Fawcett, 2006, Robin et al., 2011)

Table 1. Summary of accuracy metrics

IV. Sensitivity analyses

A. Simulation

A simulation study is induced to investigate how covariates affect model performance given current variance-covariance (var-cov) structure. We simulate data set under the true model for different scenarios. Specifically, they are described as: i) Scenario 1, all coefficients are set to 1; ii) Scenario 2, all coefficients are set to 0.1; iii) Scenario 3, all coefficients are set to 10. As shown in **Error! Reference source not found.**, we applied datasets to four models with 1000 replications. The mis-specified model denotes a model with a totally different var-cov structure.

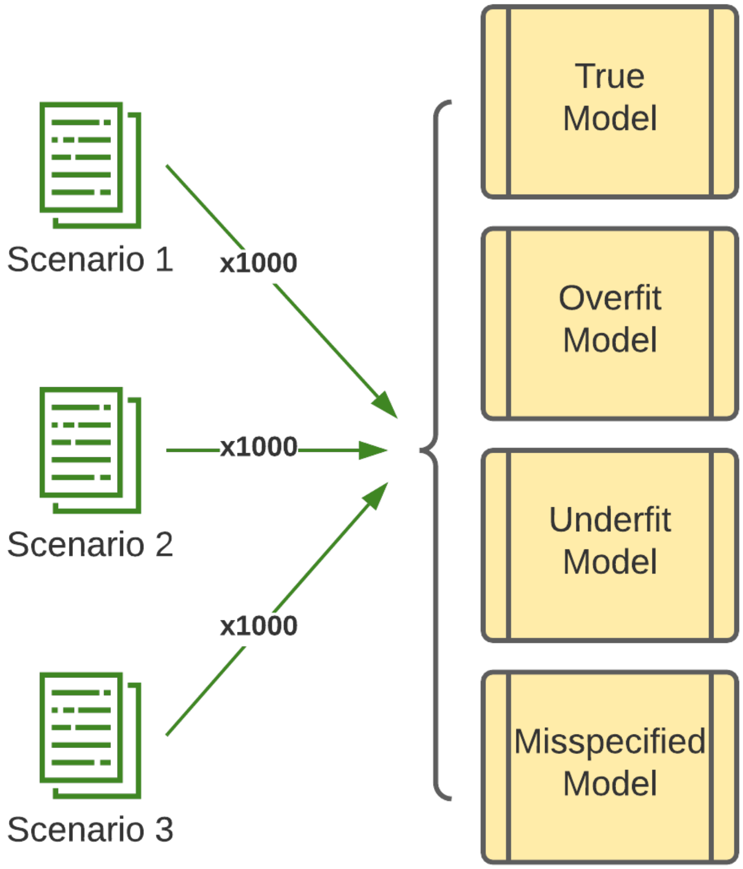

Figure 3.Simulation process

	FIT							
	TRUE		OVERFIT		UNDERFIT		MISSPECIFIED	
	RMSE (SD)	% Selected by BIC	RMSE (SD)	% Selected by BIC	RMSE (SD)	% Selected by BIC	RMSE (SD)	% Selected by BIC
**Scenario 1**	0.24 (0.01)	99.9	0.24 (0.01)	0.1	0.73 (0.02)	0	20.9 (1.44)	0
**Scenario 2**	0.24 (0.01)	100	0.24 (0.01)	0	0.25 (0.01)	0	20.95 (1.50)	0
**Scenario 3**	0.24 (0.01)	100	0.24 (0.01)	0	9.40 (0.20)	0	20.92 (1.51)	0

Table 2. Summarized results from three scenarios

B. Alternative models

Two alternative models are conducted: i) Treatment (M_2): replace change point with modulator indicator (e.g., Vx770) based on main model; ii) Birth year (M_3): replace birth cohort with centered birth year based on main model.

Model information criteria are summarized in the below Table and the smaller AIC/BIC indicates better fit. Likelihood ratio tests are aimed to assess the goodness of fit of two competing models based on the ratio of their likelihoods. The significant p-value (e.g., $p<0.05$) indicates better goodness of fit from complex model compared to nested model.

1. Model information criteria

Model	AIC[1]	BIC[2]
**Null (M_0)**	251244	251370.7
**Change point (M_1)**	250621.1	250790
**Treatment (M_2)**	250675.8	250844.7
**Birth Year (M_3)**	250612	250764

Table 3. Model comparisons

2. Likelihood ratio test

[1] Akaike information criterion
[2] Bayesian information criterion

Nested Model	Complex Model	Ddf[3]	Chisq[4]	Pr(>Chisq)[5]
M_0	M_1	5	632.8652	<0.001
M_0	M_2	5	578.171	<0.001
M_0	M_3	3	637.9623	<0.001

Table 4. Likelihood ratio test

V. Example Code

```
# LOAD PACKAGES

library(lmenssp)
library(dplyr)
library(pROC)
library(DescTools)

# FUNCTIONS

## ACCURACY.FEV1
ACCU.FEV1<-function(formula,object,filter.object,data,decimal=2){

  mf <- model.frame(formula = formula, data = data)
  y  <- as.matrix(model.extract(mf, "response"))
  x  <- as.matrix(model.matrix(attr(mf, "terms"), data = mf))

  ranef=data.frame(filter.object$u) %>%
rename(eDWID=id,u.mean=mean,u.var=variance)
  ibm=data.frame(filter.object$w) %>%
rename(eDWID=id,age=time,w.mean=mean,w.var=variance)

  out.data <- data %>%
    left_join(ranef,by='eDWID') %>%
    left_join(ibm,by=c('eDWID','time')) %>%
    mutate(fix.est=x %*% head(object$estimate[,'Estimate'],-3),
           fitted=fix.est+u.mean+w.mean,
           fitted.UCI=fitted+qnorm(0.975)*sqrt(object$estimates["tausq",1]),
           fitted.LCI=fitted-qnorm(0.975)*sqrt(object$estimates["tausq",1]))

  resid<-out.data$fitted - out.data$FEV1
  resid.rate=resid/out.data$FEV1

  RMSE = sqrt(mean(resid**2))
  MAE=sum(abs(resid))/length(resid)

  return(list(out.data=out.data,
              RMSE=round(RMSE,decimal),
```

[3] Difference of degree of freedoms
[4] Chi square statistics
[5] p-value

```
                MAE=round(MAE,decimal)
  )

}


## ACCURACY.FIES
ACCU.FIES<-function(data,decimal=2){

  data.new1 <- data %>%
    filter(!is.na(FIES.NUM)) %>%
    mutate(fitted0=fix.est+u.mean,
           threshold=ifelse(roll.base>=50,0.9*roll.base-
fitted0,0.95*roll.base-fitted0),
           prob=pnorm(threshold,w.mean,sqrt(w.var)),
           drop.ppct=1-fitted/roll.base,
           FIES.PRED=ifelse(roll.base>=50 &
drop.ppct>0.1,1,ifelse(roll.base<50 & drop.ppct>0.05,1,0)))

     #ROC-AUC
     ROC <- pROC::roc(response=data.new1$FIES.NUM, predictor=data.new1$prob)

     AUC.LCI=round(pROC::ci(ROC)[1],decimal)
     AUC=round(pROC::ci(ROC)[2],decimal)
     AUC.UCI=round(pROC::ci(ROC)[3],decimal)

     #The lower the Brier score, the better the predictions are calibrated
     BRIER=DescTools::BrierScore(resp=data.new1$FIES.NUM,pred=data.new1$prob)

     out.data <- data.new1 %>%  dplyr::select(all_of(names(data)),

prob,threshold,drop.ppct,FIES.PRED)

     return(list(
           out.data=out.data,
           BRIER=round(BRIER,decimal),
           AUC=round(AUC,decimal),
           AUC.LCI=round(AUC.LCI,decimal),
           AUC.UCI=round(AUC.UCI,decimal)))
}


# MODEL FIT
formula  <-  FEV1 ~
time*chpt+basefev1+baseage+numPEyr+numVisityr+pa+MRSA+cfrd2+Gender+isOnEnzyme
s+SESlow+bcohor

MODEL.PROPOSED <- lmenssp(formula = formula, data = in.train,
                          id = in.train$eDWID, process = "ibm",
                          timeVar = in.train$time,silent = T)

MODEL.ESTIMATE <- data.frame(MODEL.PROPOSED$estimate) %>%
                      tibble::rownames_to_column(var='Parameter') %>%
                      mutate(UCI=round(Estimate+1.96*Standard.error,2),
                             LCI=round(Estimate-1.96*Standard.error,2)) %>%
                      transmute(Parameter, Estimate,`95%CI`=paste0('(',LCI,',
                  ',UCI,')'), Standard.error,p.value)
```

```r
# AIC/BIC

MLL<-MODEL.PROPOSED$maxloglik
k <- nrow(MODEL.PROPOSED$estimates)
N <- nrow(in.train)

MODEL.AIC=2*k-2*MLL
MODEL.BIC=-2*MLL + log(N) * k

# PREDICTION METRICS
## FIT (TRAIN)
MODEL.FIT <- filtered(formula=formula,
                data = in.train,
                id = in.train$eDWID,
                process = "ibm",
                timeVar = in.train$time,
                estimate = MODEL.PROPOSED$estimate[,'Estimate'],
                subj.id = unique(in.train$eDWID))

FIT.FEV1=ACCU.FEV1(formula,object=MODEL.PROPOSED,
                   filter.object=MODEL.FIT,data=in.train)

    print(FIT.FEV1$RMSE)
    print(FIT.FEV1$MAE)


FIT.FIES=ACCU.FIES(FIT.FEV1$out.data)

    print(FIT.FIES$BRIER)
    print(FIT.FIES$AUC)
    print(FIT.FIES$AUC.LCI)
    print(FIT.FIES$AUC.UCI)


## PREDICT (TEST)
MODEL.PRED <- filtered(formula=formula,
                data = in.test,
                id = in.test$eDWID,
                process = "ibm",
                timeVar = in.test$time,
                estimate = MODEL.PROPOSED$estimate[,'Estimate'],
                subj.id = unique(in.test$eDWID))

PRED.FEV1=accuracy.FEV1(formula,object=MODEL.PROPOSED,
                        filter.object=MODEL.PRED,data=in.test)

    print(PRED.FEV1$RMSE)
    print(PRED.FEV1$MAE)

PRED.FIES=accuracy.FIES(PRED.FEV1$out.data)

    print(PRED.FIES$BRIER)
    print(PRED.FIES$AUC)
    print(PRED.FIES$AUC.LCI)
    print(PRED.FIES$AUC.UCI)
```

```
## FORECAST (MASK)
in.data=rbind(in.train,in.mask) %>% arrange(eDWID,age)

MODEL.FRCT <- smoothed(formula=formula,
                 data = in.data,
                 id = in.data$eDWID,
                 process = "ibm",
                 timeVar = in.data$time,
                 estimate = MODEL.PROPOSED$estimate[,'Estimate'],
                 subj.id = unique(in.data$eDWID))

FRCT.FEV1=ACCU.FEV1(formula,object=MODEL.PROPOSED,
                    filter.object=MODEL.FRCT,data=in.data)

    print(FRCT.FEV1$RMSE)
    print(FRCT.FEV1$MAE)

FRCT.FIES=ACCU.FIES(FRCT.FEV1$out.data)

    print(FRCT.FIES$BRIER)
    print(FRCT.FIES$AUC)
    print(FRCT.FIES$AUC.LCI)
    print(FRCT.FIES$AUC.UCI)
```

Online supplement (Part 2)

1 Mixed effects with nonstationary stochastic process model

Let Y_{ij} denote longitudinal measurement (FEV$_1$) for the ith patient taken at age t_{ij}, $i = 1, \ldots, n, j = 1, \ldots, n_i$. Our model can be expressed in the form of,

$$Y_{ij} = \mu_i(t_{ij}) + U_i + W_i(t_{ij}) + Z_{ij}, \tag{1}$$

where $\mu_i(t_{ij}) = f(t_{ij}) + \boldsymbol{X\alpha}$ represents nonlinear interpolation in FEV$_1$ trajectories at population level. $\boldsymbol{X}$ denotes a design matrix including both static and time-varying covariates and $\boldsymbol{\alpha}$ is the corresponding coefficient. $f(\boldsymbol{t})$ is truncated cubic splines with five knots ($k_1 = 10.95, k_2 = 14.94, k_3 = 18.97, k_4 = 24.84, k_5 = 33.15$) at age in years computed by the quantile method in [3]. Random intercept U_i represents between-patient heterogeneity with an assumption of $U_i \sim N(0, \omega^2)$. Measurement error is independent and identically distributed random variables, such that $Z_{ij} \sim N(0, \tau^2)$. The term W_{ij} denotes a stochastic process, it could be either stationary (e.g., powered correlation function, Matern correlation function, etc.) or nonstationary (e.g., Brownian motion, integrated Brownian motion, integrated Ornstein-Uhlenbeck process, etc.). Herein, we specify $W_i(t)$ as integrated Brownian motion such that $W_i(t) = \int_0^t B_i(v)dv$, where $B_i(v)$ is the rate of change in lung function at time v depicted as Brownian motion. We assume $B_i(t) \sim N(0, \sigma^2 t)$, $Cov(B_i(s), B_i(t)) = \sigma^2 min(s,t)$ and $B_i(0) = 0$. It further turns out that $\boldsymbol{W}_i \sim N(\boldsymbol{0}, \boldsymbol{R}_i)$, where $\boldsymbol{R}_i$ is a $n_i \times n_i$ variance-covariance matrix with covariance function for age s and t as

$$Cov\big(W_i(s), W_i(t)\big) = \sigma^2 \frac{min(s,t)^2}{2}\Big(max(s,t) - \frac{min(s,t)}{3}\Big). \tag{2}$$

2 Target function

Predictive probability function (also called target function) of FIES according to its definition is described as

$$\begin{aligned} Pr(R_{ij} = 1|\boldsymbol{D}_{obs}) &= Pr(Y_{ij} < \delta_{ij}|\boldsymbol{D}_{obs}) \\ &= Pr(\widehat{W}_{ij} < \delta_{ij} - \hat{\mu}_{ij} - \hat{U}_i|\boldsymbol{D}_{obs}) \\ &= \Phi\Big(\frac{\delta_{ij} - \hat{\mu}_{ij} - \hat{U}_i - \hat{\mu}_{w_{ij}}}{\hat{\sigma}_{w_{ij}}}\Big), \end{aligned} \tag{3}$$

where $\boldsymbol{D}_{obs} = (\boldsymbol{B}_k, \boldsymbol{X}_{ij})^T$, Φ represents standard normal distribution $N(0,1)$, individual threshold of FIES $\delta_{ij} = 0.9 \times C_{ij} I(C_{ij} \geq 50) + 0.95 \times C_{ij} I(C_{ij} < 50)$ and C_{ij} denotes baseline FEV$_1$ in prior year. $\boldsymbol{\mu}_{ij}$ and U_i are as before. $\mu_{w_{ij}}$ and $\sigma_{w_{ij}}$ denote mean and standard deviation for $W_i(t_{ij})$. As these parameters are not observed, we replace them with their MLEs ((see section 4.1 in [2] for MLE details)).

In order to achieve 95% confidence interval (CI) for the predictive probability, we apply simulation-based bootstrapping algorithm as previously implemented in [4]. Let $\boldsymbol{\mu}_{w_i}$ and $\boldsymbol{\Sigma}_{n_i}$ denote the mean

and covariance matrix for $\boldsymbol{W}_i$. We draw independent $L = 100$ samples for each patient at time t_{ij} via following steps:
1. Let $Q_l \sim N(\boldsymbol{0}, \widehat{\boldsymbol{\Sigma}}_{n_i})$, where $l = 1, \ldots, L$ and $\widehat{\boldsymbol{\Sigma}}_{n_i}$ is the estimate of $\boldsymbol{\Sigma}_{n_i}$ from the proposed model.
2. Compute $\boldsymbol{\mu}^*_{w_i} = \hat{\boldsymbol{\mu}}_{w_i} + (\sqrt{n_i})^{-1} Q_l$ by the key assumption of $\sqrt{n_i}(\hat{\boldsymbol{\mu}}_{w_i} - \boldsymbol{\mu}_{w_i}) \xrightarrow{D} N(\boldsymbol{0}, \boldsymbol{\Sigma}_{n_i})$.
3. Given $\boldsymbol{\mu}^*_{w_i} = (\mu^*_{w_{i1}}, \ldots, \mu^*_{w_{ij}})^T$ with $j = 1, \ldots, n_i$, calculate $\Phi\big(\frac{\delta_i - \hat{\mu}_{ij} - \hat{U}_i - \hat{\mu}^*_{w_{ij}}}{\hat{\sigma}_{w_{ij}}}\big)$ derived in Equation (3).
4. Compute mean squared error $\text{MSE} = L^{-1}\sum_{l=1}^{L}\Big(\Phi\big(\frac{\delta_{ij} - \hat{\mu}_{ij} - \hat{U}_i - \hat{\mu}^*_{w_{ij}}}{\hat{\sigma}_{w_{ij}}}\big) - \Phi\big(\frac{\delta_{ij} - \hat{\mu}_{ij} - \hat{U}_i - \hat{\mu}_{w_{ij}}}{\hat{\sigma}_{w_{ij}}}\big)\Big)^2$.
5. Construct $(1-\alpha) \times 100\%$ CI for predictive probability as $Pr(R_{ij} = 1|\boldsymbol{D}_{obs}) \pm z_{1-\alpha/2}\sqrt{\text{MSE}}$ with $\alpha = 0.05$ and z denoting z score of standard normal distribution.

3 Predictions

3.1 Population Level

Equation (1) induces multivariate normal distribution for Y_i as

$$\boldsymbol{Y}_i \sim N(\boldsymbol{\mu}_i, \boldsymbol{V}_i(\boldsymbol{\phi})), \tag{4}$$

where $\boldsymbol{Y}_i = (Y_{i1}, \ldots, Y_{in_i})^T$, $\boldsymbol{V}_i(\boldsymbol{\phi})$ can be decomposed as

$$\boldsymbol{V}_i(\boldsymbol{\phi}) = \omega^2 \boldsymbol{J}_i + \sigma^2 \boldsymbol{R}_i + \tau^2 \boldsymbol{I}_i, \tag{5}$$

where $\boldsymbol{\phi} = \{\omega^2, \sigma^2, \tau^2\}$, $\boldsymbol{J}_i$ is an $n_i \times n_i$ matrix of ones, $\boldsymbol{R}_i$ is as before and $\boldsymbol{I}_i$ is an $n_i \times n_i$ identity matrix.

We express expected value of FEV_1 at time t at population level as

$$E(\boldsymbol{Y}_{i*}) = \boldsymbol{\mu}_{i*} = \beta_0 + \sum_{k=1}^{K} \beta_k \boldsymbol{B}_k(t) + \boldsymbol{X}_{i*}\boldsymbol{\alpha}, \tag{6}$$

where $i*$ denotes the subject with averaged covariates, particularly, we take the mean of continuous covariates and the mode of categorical covariates based on the change point. $\boldsymbol{B}_k$ denotes truncated cubic splines basis vector at kth location and β_k is the corresponding coefficient. β_0 is an overall intercept such that $\beta_0 + \sum_{k=1}^{K} \beta_k \boldsymbol{B}_k(t)$ measure the nonlinear fixed effect over time. $\boldsymbol{X}_{i*}$ is the design matrix with averaged covariates and $\boldsymbol{\alpha}$ is as before. In order to calcuate averaged FEV_1 against entire observed period, we plug in maximum likelihood estimates (MLEs) of β_0, β_k and $\boldsymbol{\alpha}$.

3.2 Individual Level

The conditional distribution $\pi(U_i|\boldsymbol{Y}_i, \boldsymbol{\theta})$, $\pi(W_i(t_{ij})|\boldsymbol{Y}_i^j, \boldsymbol{\theta})$ and $\pi(W_i(t_{ij}+u)|\boldsymbol{Y}_i^j, \boldsymbol{\theta})$ are of importance for individual's prognosis, with $\boldsymbol{Y}_i^j = (Y_{i1}, \ldots, Y_{ij})^T; \boldsymbol{\theta} = \{\boldsymbol{\alpha}, \boldsymbol{\beta}, \boldsymbol{\phi}^T\}$; lead-time u. We illustrate individual dynamic prediction by properties of multivariate normal distribution ([2], [1]) as follows,

$$\text{Nowcasting for new patient } i'\text{: } E(Y_{i'j}) = E(\boldsymbol{\mu}_{i'}(t_{i'j})|\boldsymbol{\theta}) + E(U_{i'}|\boldsymbol{Y}_{i'}, \boldsymbol{\theta}) + E(W_{i'}(t_{i'j})|\boldsymbol{Y}_{i'}^j, \boldsymbol{\theta}); \tag{7}$$

$$\text{Forecasting for new time point } j'\text{: } E(Y_{ij'}) = E(\boldsymbol{\mu}_i(t_{ij}+u)|\boldsymbol{\theta}) + E(U_i|\boldsymbol{Y}_i, \boldsymbol{\theta}) + E(W_i(t_{ij}+u)|\boldsymbol{Y}_i^j, \boldsymbol{\theta}). \tag{8}$$

Substitute all unknown parameters with their MLEs in Equation (7) & (8) as,

$$\text{Nowcasting}\begin{cases} E(\boldsymbol{\mu}_{i'}(t_{i'j})|\hat{\boldsymbol{\theta}}) = \hat{\beta}_0 + \sum_{k=1}^{K} \hat{\beta}_k \boldsymbol{B}_k(t_{i'j}) + \boldsymbol{X}_{i'j}\hat{\boldsymbol{\alpha}}; \\ E(U_{i'}|\boldsymbol{Y}_{i'}, \hat{\boldsymbol{\theta}}) = \hat{\omega}^2 \boldsymbol{J}_{n_i',1}^T \widehat{\boldsymbol{V}}_{i'}^{-1}(\boldsymbol{Y}_{i'} - \hat{\boldsymbol{\mu}}_{i'}); \\ E(W_{i'}(t_{i'j})|\boldsymbol{Y}_{i'}, \hat{\boldsymbol{\theta}}) = \frac{\hat{\sigma}^2}{2}\boldsymbol{F}_{i'}^{j^T}(\widehat{\boldsymbol{V}}_{i'}^j)^{-1}(\boldsymbol{Y}_{i'}^j - \hat{\boldsymbol{\mu}}_{i'}^j), \end{cases} \tag{9}$$

$$\text{Nowcasting}\begin{cases} E(\boldsymbol{\mu}_i(t_{ij}+u)|\hat{\boldsymbol{\theta}}) = \hat{\beta}_0 + \sum_{k=1}^{K} \hat{\beta}_k \boldsymbol{B}_k(t_{ij}+u) + \boldsymbol{X}_{ij}\hat{\boldsymbol{\alpha}}; \\ E(U_i|\boldsymbol{Y}_i, \hat{\boldsymbol{\theta}}) = \hat{\omega}^2 \boldsymbol{J}_{n_i,1}^T \widehat{\boldsymbol{V}}_i^{-1}(\boldsymbol{Y}_i - \hat{\boldsymbol{\mu}}_i); \\ E(W_i(t_{ij}+u)|\boldsymbol{Y}_i^j, \hat{\boldsymbol{\theta}}) = \frac{\hat{\sigma}^2}{2}\boldsymbol{F}_i^{j,u^T}(\widehat{\boldsymbol{V}}_i^j)^{-1}(\boldsymbol{Y}_i^j - \hat{\boldsymbol{\mu}}_i^j), \end{cases} \tag{10}$$

where $\boldsymbol{F}_i^j = (t_{i1}^2(t_{ij} - t_{i1}/3), \ldots, t_{ij}^2(t_{ij} - t_{ij}/3))^T$, $\boldsymbol{F}_i^{j,u} = (t_{i1}^2(t_{ij}+u-t_{i1}/3), \ldots, t_{ij}^2(t_{ij}+u-t_{ij}/3))^T$, $\boldsymbol{J}_{n_i,1}$ (or $\boldsymbol{J}_{n_i',1}$) is $n_i \times 1$ (or $n_i' \times 1$) matrix of ones, $\boldsymbol{V}_i^j$ is the variance-covariance matrix of $\boldsymbol{Y}_i^j$.

References

[1] T.W. Anderson. *An Introduction to Multivariate Statistical Analysis, 2nd edition.* John Wiley & Sons., New York, 1984.

[2] PJ Diggle, I Sousa, and O Asar. Real-time monitoring of progression towards renal failure in primary care patients. *Biostatistics*, 16(3):522–536, 2015.

[3] L. Ngo and M. P. Wand. Smoothing with mixed model software. *Journal of Statistical Software*, 9(1):1–54, 2004.

[4] R.D. Szczesniak, W. Su, C. Brokamp, H.K. Ruth, J.P. Pestian, M. Seid, P.J. Diggle, and J.P. Clancy. Dynamic predictive probabilities to monitor rapid cystic fibrosis disease progression. *Statistics in Medicine*, 39:740–756, 2020.
